# Gender differences in the comprehension of care plans in an emergency department setting

**DOI:** 10.1186/s13584-018-0245-9

**Published:** 2018-09-18

**Authors:** Shachaf Shiber, Rona Zuker-Herman, Michael J. Drescher, Marek Glezerman

**Affiliations:** 1 The Department of Emergency Medicine, Rabin Medical Center, Beilinson Campus, 49100 Petach-Tikva, Israel; 2 Sackler Faculty of Medicine, Tel Aviv University, Tel Aviv-Yafo, Israel; 3 Research Center for Gender Medicine, Rabin Medical Center, Beilinson Campus, Petah Tikva, Israel

**Keywords:** Gender medicine, Plan of care, Ethnic differences, Comprehension

## Abstract

**Background:**

Previous studies have shown that, in a variety of health care settings, patients often do not understand what health care professionals tell them about their diagnoses and care plans; this is particularly true among male patients. Emergency department (ED) settings present unique challenges to communication with patients due to the rapid pace of activity, substantial changes in personnel over the course of the day and the week, and the need for fast decision-making processes. The aim of our study was to investigate the extent to which patients in an Israeli ED comprehended their plan of care and whether there were gender differences in this regard.

**Methods:**

We conducted a questionnaire-based prospective study, in which patients admitted to the ED at Rabin Medical Center were evaluated during the years 2014–2016. The primary outcome was patients’ comprehension of their plan of care, stratified by gender of patients. Plan of care included information related to diagnosis, treatment and discharge instructions. The secondary outcome was patients’ satisfaction with the instruction process.

**Results:**

One hundred seventy seven ED patients met study criteria and were asked to participate in the study; 85% of them agreed to do so. Overall, 150 ED patients aged 18–80 were recruited [75 men (50%) and 75 women (50%)]. 80% of the respondents reported a satisfactory understanding of their plan of care. Overall, no gender-related differences were found. Differences between men and women concerning satisfaction with the instructions provided by nurses were found among non-Hebrew speakers, but not among Hebrew speakers.

**Conclusion:**

Contrary to most earlier studies, patients at our ED demonstrated a high degree of self-reported adequate comprehension concerning their plan of care, and overall no gender-related differences were found. These finding may be due in part to improved training of the medical staff to better communicate with the patients and to answer their questions. In addition, patients may feel more comfortable than in the past about asking the medical staff questions regarding their plan of care and diagnosis. The main implication of this study is that physician education programs should continue to emphasize patient-physician communications skills and improving methods for providing patients with information.

## Background

Good communication between health care professionals and their patients is fundamental for ensuring comprehension and providing effective care. Studies in ambulatory and emergency settings document that appropriate communication between patients and physicians regarding the diagnosis and treatment plan is associated with improved medication adherence and better outcomes (Kerse, et al. 2004; [1–3]).

Prior studies have revealed that most hospitalized patients are unable to correctly identify their physicians or nurses [4–6].

At hospital discharge, patients are frequently unable to list their diagnoses and the names, purposes, and adverse effects of medications [7, 8]. Moreover, physicians frequently overestimate patients’ understanding of their treatment plans at the time of hospital discharge [7] and may not recognize patients with low health literacy [9].

Emergency department (ED) settings present unique challenges to patient-physician interaction, given the lack of a prior physician-patient relationship, the rapid pace of clinical care, and the dynamic nature of the working patterns of the medical teams. Alberti and Nannini [3], reviewed twenty-one studies (including children and adults) that looked comprehension of discharge instructions in the ED. They could identify only 4 randomized controlled trials and observed that clinicians rarely verify patients’ comprehension and that the current used methodology may not be satisfactory. Moreover, none of the evaluated studies addressed gender aspects of patient’s comprehension of instructions received.

A small number of studies have reported on differences in physician-patient interactions and assessments of comprehension of care plans in associated with patients’ gender in a variety of health care settings [10–13]. In 2011, Safdar et al. [14] assessed the extent of the effect of gender on health outcomes in published emergency medicine (EM) studies. They claimed that emergency physicians, as front-line clinical specialists, may directly advance patient care by understanding how gender-specific approaches may affect the evaluation and management of diseases in an acute setting. The authors searched MEDLINE for a 3.3-year study period in order to provide insight related to the inclusion of gender in EM research. Most of the research articles listed gender as a demographic variable, but only 18% studied the independent effect of gender on health outcomes.

Gender differences may influence communication within the medical encounter between medical staff and patients. Nevertheless, gender aspects in this context are still a largely ignored issue. Carpenter et al. [15] studied 161 women and 71 men with vasculitis. The authors reported that the most pronounced difference in information seeking was related to gender. They found that women searched the internet more often than men, whereas men were more likely than women to consult health professionals.

In Singapore (Heng, et al.), a study of 110 patients in the ED with closed-head injury found that 29% of patients who received head injury advice were non-compliant with instructions. Males were less likely to recall their discharge instructions (Mean recall scores - female 2.3 versus male 1.6, *p* = 0.024)).

The aim of our study was to investigate the extent to which patients in an Israeli ED comprehended their plan of care and whether there were gender differences in this regard. We set out with two hypotheses. First, we hypothesized that many patients do not sufficiently understand treatment plans and discharge instructions, mostly due to the stressful setting in an emergency ward. The second hypothesis was that women would be more likely than men to understand proposed treatment plans and discharge instructions in an ED setting, as women are more ‘health conscious’ than men, ask more health-related questions and use more resources to obtain health-related information.

## Methods

### Study population

This was a questionnaire-based prospective study in which patients aged 18–80 admitted to the ED at the Rabin Medical Center during the years 2014–2016 were evaluated.

Rabin Medical Center is a tertiary health care located in central Israel, and its ED is the largest in Israel, with over 150,000 annual visits.

During the study period, the patients were recruited randomly by the authors. Recruitment occurred only during day shifts at which at least one of the authors was present in the ED. We intended to recruit consecutively each 5th to 10th patient discharged. Due to technical constraints, we recruited only once every week or two, choosing every 5th to 10th h patient discharged, and repeating the process up to three times a day. This may assist in explaining our slow recruitment.

In order to be included in the study, patients needed to be over 18 years of age, understand the consent form and be able and willing answer the questionnaire When Hebrew was not the patient’s native language (Arabs and native Russian speakers) we provided forms in different languages and translator services.

Patients were requested to answer demographic data and questions related to their understanding of their ED plan of care. Informed consent was received from all patients before enrolment. We used our comprehensive medical electronic data system for confirmation of demographic data.

The study was approved by the Rabin Medical Center Institutional Review Board.

An identical gender-neutral questionnaire for women and men was developed specifically for the study that was partially based on an earlier field study ([16], Appendix 1). We noted that Alberti and Nannini [3], in their extensive review of twenty one studies, reported that 71% of the included studies were also based on discharge interviews and a quiz regarding discharge instructions.

The questionnaire was reviewed for face and content validity, according to the recommendation of Sangoseni et al. [17]. Content validity of a questionnaire can be established by using a panel of experts in the field of the relevant questionnaire. We used a panel of three emergency physicians and three emergency nurse, all experts in this field. They reviewed the questionnaire items for readability and comprehensibility; they also agreed on which questions should be included in the final questionnaire. The reviews were performed in a dichotomous manner, i.e. an item is either accepted or rejected. Face validity was assessed by our expert group and by our statistician who reviewed the final list of selected questions. We consider a positive interaction if the patients study answered the question with a score greater than 3 (i.e. either “quite a bit” or “completely”) on a 5 point scale with the following options: not at all, a little bit, moderately, quite a bit, completely.

### Statistics

Descriptive statistics were used for the analysis. Categorical variables were presented numerically as a percentage of total patients in the cohort (N, %), and Fisher’s exact test was used to compare the values of the categorical variables, with statistical significance defined as *P* < 0.05, with Bonferroni correction for the number of items. Estimation of the sample size required for the study was based on the expected difference in gender comprehension plan of care. It was hypothesized that a minimum absolute difference of 10% was important to detect difference between genders. Using a power of 80% and an alpha of 5%, a required sample size of 112 patients calculated.

The statistical analysis for this paper was generated using SAS Software, Version 9.4.

## Results

The study consisted of 150 patients aged 18–80 years who were admitted to the ED during the study period with a veriety aetiologies. Only patients who were able to understand and sign the consent form were included (translation services were available for the consent process).

Overall, we asked for the participation of 177 patients who were compatible with the study inclusion criteria, and 27 of them refused to answer or complete the questionnaire. Thus, the response rate was 85%.

Eleven patients refused to participate in the study due to a lack of time, 10 patients felt the questionnaire was too long and stopped after the first two questions, and 6 patients did not understand the questions in the questionnaire. None of the partially filled questionnaires was useable for analysis.

The epidemiological data of patients who did not complete the questionnaire compared to those of the study population is listed in Appendix 2.

As mentioned, 150 ED patients were recruited (75 men and 75% women). The background characteristics of patients (demographics, experience with ED) are presented in Table 1. 80% of the patients reported a satisfactory understanding of their plans of care and 76% of their discharge instructions. 53% had previously signed informed consent documents. The overall understanding of the plan of care (above 3 points) from the nurses was 84% and from the physicians was 95%. Most of the patients (91%) were asked by their ED physician during their visit in the ED, if they agreed to the treatment plan, and the majority (85%) received their discharge instructions from a physician. (Table 2).

**Table 1:** Background characteristics of patients

	Women (*n* = 75)	Men (*n* = 75)	Overall (*n* = 150)	*P*-value
Number of patients (n, %)	75(50%)	75(50%)	150(100%)	
Age Group (mean ± SD)	49.5 ± 19.73	45.96 ± 21.55	47.4 ± 20.48	0.29
18–24 (n, %)	7 (9.3%)	11 (14.6%)	18 (12%)	0.34
25–54 (n, %)	14 (18.6%)	15 (20%)	29 (19.3%)	
35–55 (n, %)	17 (22.6%)	23 (30.6%)	40 (26.7%)	
> 55 (n, %)	37 (49.3%)	26 (34.6%)	63 (42%)	
Education				0.23
Elementary school (n, %)	3 (4%)	7 (9.3%)	10 (6.7%)	
High school (n, %)	40 (53.3%)	34 (45.3)	74 (51.3%)	
Academic degree (n, %)	32 (42.6%)	31 (41.3%)	63 (42%)	
Ph.D. (n, %)	0	3 (4%)	3 (2%)	
Native Language				0.47
Hebrew (n, %)	50 (66.7%)	40 (53.3%)	90 (60%)	
Arabic (n, %)	10 (13.3%)	17 (22.6%)	27 (18%)	
Russian (n, %)	11 (14.6%)	9 (12%)	20 (13.3%)	
Other (n, %)	4 (5.3%)	9 (12%)	13 (17.3%)	
Type of referral				0.37
Trauma (n, %)	6 (8%)	14 (18.6%)	20 (13.3%)	
Surgical (n, %)	8 (10.6%)	10 (13.3%)	18 (12%)	
Internal (n, %)	40 (53.3%)	30 (40%)	70 (46.6%)	
Neurologic (n, %)	8 (10.6%)	7 (9.3%)	15 (10%)	
Orthopaedic (n, %)	13 (17.3%)	14 (18.6%)	27 (18%)	
Visit to the ED				0.51
Number of visits to the ED in the last 5 years (mean + SD)	1.76 ± 1.41	1.86 ± 1.68	1.81 ± 1.54	
Number of visits to the ED for other complaints in the last 5 years (mean + SD)	1.08 ± 1.21	1 ± 1.24	1.04 ± 1.22	
Number of visits with other complaints to the ED in the last 5 years (mean + SD)	0.63 ± 0.89	0.81 ± 1.32	0.72 ± 1.12	
Accompanying person in the ER (n, %)	45 (60%)	50 (66.67%)	95 (63.3%)	0.5

**Table 2:** Positive answers of important question concerning care plans in the ED (scores > 3 on a 5 point scale)

	Women (n = 75)	Men (n = 75)	Overall (n = 150)
After the conversation with the nurses, I know more about my condition	67 (89%)	59 (79%)	126 (84%)
After the conversation with the physician, I know more about my condition	72 (96%)	70 (93%)	142 (95%)
The nurse explained the ED process to me	54 (72%)	49 (65%)	113 (75%)
The physician explained the ED process to me	70 (93%)	69 (92%)	139 (93%)
The medical staff explained the instructions at discharge to me	65 (86%)	61 (81%)	126 (84%)
I understand the treatment process in the ED	65 (86%)	56 (75%)	121 (80%)
I understand the discharge instructions	58 (77%)	57 (76%)	115 (76%)
I know what to do about my medical situation after discharge	59 (79%)	57 (76%)	116 (76%)
I understand my diagnosis	61 (81%)	63 (85%)	124 (83%)

We stratified the data by age, education, native language (Hebrew and other), referral type, experience with the informed consent process and medical knowledge (subjective). No differences among these groups were found in patients’ understanding of their plans of care or discharge instructions. Additionally, no differences were found among these groups regarding satisfaction with nurses and physicians with how they provided informations about their plan of care, and the way medical staff performed in the ED. Overall, no gender-related differences were found. Differences between men and women concerning satisfaction with the instructions provided by nurses were found only among non-Hebrew speakers, and but not among Hebrew speakers, Table 3 displays the data on these differences.

**Table 3:** Unsatisfactory interactions with nurses, by language group and gender(scores of less than 3 on a 5 point scale)

	Hebrew native language speaker		*P*-value	Other native language speaker		*P*-value
	Women (*n* = 50)	Men (*n* = 40)		Women (*n* = 25)	Men (*n* = 35)	
After the conversation with the nurses, I know more about my condition	5/50 (10%)	6/40 (15%)	0.47	3/25 (12%)	20/35 (57%)	*P* < 0.001
I am satisfied with the nurses’ treatment	6/50 (12%)	7/40 (18%)	0.5	4/25(16%)	22/35 (63%)	P < 0.001
The nurses explained the ED process to me	5/50(10%)	5/40(13%)	0.45	4/25(16%)	20/35 (57%)	*P* = 0.001

## Discussion

In this study, the results did not confirm our baseline hypotheses. Over 75% of patients understood both treatment plans and discharge instructions adequately; we also did not find a gender differential in patients’ comprehension in this context. The only exception was with male patients who were not native Hebrew speakers; they were less satisfied with the explanations about their plan of care received from nurses as opposed to those received from physicians. As our medical staff includes both women and men physicians, this preference appears to be status-based, rather than gender-based.

The main study findings are contrary to those of many previous studies which found low levels of patient comprehension of provider communications and particularly low levels among male patients. Possible explanations for these somewhat unique findings include the presence of a well-qualified medical team due to improved educational programmes regarding communication physicians-patients in Israeli medical schools. Also, in this particular hospital there are annual workshops for medical staff with simulations of patient-physician and patient−/nurse interactions, with feedback from the audience and the instructor.

There are a number of limitations to our study. Firstly, the questionnaire was presented to the patients at discharge. Taking into account that the ED is a stressful environment with long waiting times for examinations, test results and treatment, it is understandable that patients may be reluctant to spend additional time filling out questionnaires. It is therefore possible that those who agreed to enrol in the study do not necessarily represent others who refused to participate.

Secondly, when we analysed responses related to satisfaction of patients, we did not use multivariate analysis due to the small number of patients. Therefore, confounding factors may have affected the result of this analysis.

Thirdly, we recruited patients only during day shifts. Therefore, our results may not be representative for patients who present at the ED during the night with the additional aspects of urgency, although those findings can represent an overall change in medical staff- patients relationships; we recommend conducting parallel studies in additional Israeli’s ED and in different clinics and department.

## Conclusion

In conclusion, we found overall good patient comprehension in the ED related to explanations and instructions, with no gender differences. These finding may be due to improved training of the medical staff to communicate better with the patients and to answer their questions. In addition, advances in digital health and social media have made it easier for patients to read and understand their discharge letters and patients feel more comfortable asking the medical staff questions about their plan of care and diagnosis. We found differences among men who were not native Hebrew speakers, who preferred to receive pertinent information from physicians rather than from nurses, but this preference may not have been gender-related but status-based. Further research may be needed to understand this tendency. This may require a qualitative based research protocol, including input from social sciences as opposed to a quantitative research protocol.

The main implication of this study is that physician education programs should continue to emphasize patient-physician communications skills and improving methods for providing patients with information.

## Appendix 1

### Questionnaire

1. Age
2. Sex
3. Number of ED visit in last 5 years
4. Education
5. Accompanying person - yes/no
6. Native language
7. Do you need translation in the ED? - yes/no (if no, jump to question 9)
8. Has a translator been provided to you? - yes/no
9. After the conversation with the nurses, I know more about my condition - not at all, a little bit, moderately, quite a bit, completely
10. After the conversation with the physician, I know more about my condition - not at all, a little bit, moderately, quite a bit, completely
11. I am satisfied with the nurses’ treatment - not at all, a little bit, moderately, quite a bit, completely
12. I am satisfied with the way the physician treated me - not at all, a little bit, moderately, quite a bit, completely
13. The nurse explained the ED process to me - not at all, a little bit, moderately, quite a bit, completely
14. The physician explained the ED process to me - not at all, a little bit, moderately, quite a bit, completely
15. The medical staff explained the instructions at discharge to me - not at all, a little bit, moderately, quite a bit, completely
16. I understand the treatment process in the ED - not at all, a little bit, moderately, quite a bit, completely
17. I understand the discharge instructions - not at all, a little bit, moderately, quite a bit, completely
18. I know what to do about my medical situation after discharge - yes/no
19. I understand my diagnosis - yes/no
20. The physician requested my consent to the treatment process - yes/no
21. Explanations were given to me in my native language - yes/no. If no: With the help of a translator - yes/no
22. Who provided discharge instructions - doctor/nurse/other
23. In the past, I have signed informed consent forms - yes/no

## Appendix 2

**Table 4:** Epidemiological data of patients who did not complete the questionnaire compared to that of the study population

	Study patients	Excluded patients	*P*-value
Number of patients (n, %)	150 (84.8%)	27 (15.2%)	
Age group (mean ± SD)	47.4 ± 20.48	50.1 + 26.1	0.31
Gender M:F	75:75	13:12	0.92
Native language			0.2
Hebrew (n, %)	90 (60%)	20 (74%)	
Arabic (n, %)	27 (18%)	5 (18.5%)	
Russian (n, %)	20 (13.3%)	2 (7.4%)	
Other (n, %)	13 (17.3%)	0	
